# Molecular details of ligand selectivity determinants in a promiscuous β-glucan periplasmic binding protein

**DOI:** 10.1186/1472-6807-13-18

**Published:** 2013-10-04

**Authors:** Parthapratim Munshi, Christopher B Stanley, Sudipa Ghimire-Rijal, Xun Lu, Dean A Myles, Matthew J Cuneo

**Affiliations:** 1 Neutron Sciences Directorate, Oak Ridge National Laboratory, Oak Ridge, TN 37831, USA; 2 Department of Chemistry, Middle Tennessee State University, Murfreesboro, TN 37132, USA; 3 Current address. Shiv Nadar University, Department of Chemistry, Oak Ridge National Laboratory, Uttar Pradesh, India

**Keywords:** Periplasmic binding protein, Carbohydrate recognition, Laminarin, ABC transport, Ligand specificity

## Abstract

**Background:**

Members of the periplasmic binding protein (PBP) superfamily utilize a highly conserved inter-domain ligand binding site that adapts to specifically bind a chemically diverse range of ligands. This paradigm of PBP ligand binding specificity was recently altered when the structure of the *Thermotoga maritima* cellobiose-binding protein (tmCBP) was solved. The tmCBP binding site is bipartite, comprising a canonical solvent-excluded region (subsite one), adjacent to a solvent-filled cavity (subsite two) where specific and semi-specific ligand recognition occur, respectively.

**Results:**

A molecular level understanding of binding pocket adaptation mechanisms that simultaneously allow both ligand specificity at subsite one and promiscuity at subsite two has potentially important implications in ligand binding and drug design studies. We sought to investigate the determinants of ligand binding selectivity in tmCBP through biophysical characterization of tmCBP in the presence of varying β-glucan oligosaccharides. Crystal structures show that whilst the amino acids that comprise both the tmCBP subsite one and subsite two binding sites remain fixed in conformation regardless of which ligands are present, the rich hydrogen bonding potential of water molecules may facilitate the ordering and the plasticity of this unique PBP binding site.

**Conclusions:**

The identification of the roles these water molecules play in ligand recognition suggests potential mechanisms that can be utilized to adapt a single ligand binding site to recognize multiple distinct ligands.

## Background

The periplasmic binding proteins (PBP) are a protein superfamily that serve as primary receptors for a diverse group of metabolic solutes in signaling [1], chemotaxis [2] and metabolite transport systems in bacteria [3], eukaryotes and archaea. PBP mediated transmembrane transport of ligands are coupled to either ATP hydrolysis (ABC transport) [4] or H^+^/M^+^ motive force (TRAP transport or tripartite tricarboxylate transport) [5]. In addition, the PBP module is also found in enzymes [6], transcriptional control elements [7] and eukaryotic neurotransmission systems [8]. PBPs bind multiple ligands that range in size from a few Daltons to as large as 1 kDa, including ions [9], amino acids [10], peptides [11], monosaccharides [12], oligosaccharides [13], polyamines [14], oxidized inorganics [15]. Many other ligands continue to be discovered through current genome sequencing technology [16].

Despite the wide variation in PBP cognate ligand size and chemical functionality, the three-dimensional structure is highly conserved across all PBPs. PBPs are comprised of two α/β domains connected by a flexible linker region that serves as a pivot point for the ligand induced hinge-bending motion that this protein superfamily is known for [17-20]. PBPs were initially classified into three distinct sub-groups based upon the topology of β-strands in each domain [21]. Recently, the PBP super-family was re-categorized into six distinct clusters by combining known ligand specificities with the wealth of structural information available in the Protein Data Bank [22].

PBPs typically bind cognate ligands with exquisite specificity, discriminating among anomeric/epimeric carbohydrates or different ions [23,24]. This remarkable ability to specifically bind their cognate ligands from pools of similarly related molecules has been attributed to the localization of the PBP ligand binding site at the interdomain interface [25]. In the apo form, ligand binds to a highly adaptable solvent exposed surface which upon complexation with ligand, and the other PBP domain, produces an environment similar to a less adaptable solvent excluded protein core [26,27]. In most cases PBP function relies on differential recognition of the apo and ligand bound forms of the protein by transmembrane-bound proteins [28]. Ligand binding at the interdomain interface stimulates a conformational change which is characterized as a rigid body hinge-bending/twisting motion about the interdomain linker region. The relative orientation of the two domains changes by as much as 60-70° [29,30], although the magnitude of the hinge-bending motion is variable and can be rather small in some cases [31]. The conformational coupling of ligand binding and function is also conserved when the PBP module is found in larger multidomain proteins, such as the eukaryotic glutamate receptor [8] and the LacI family of transcriptional regulators [7].

**Figure 1:**
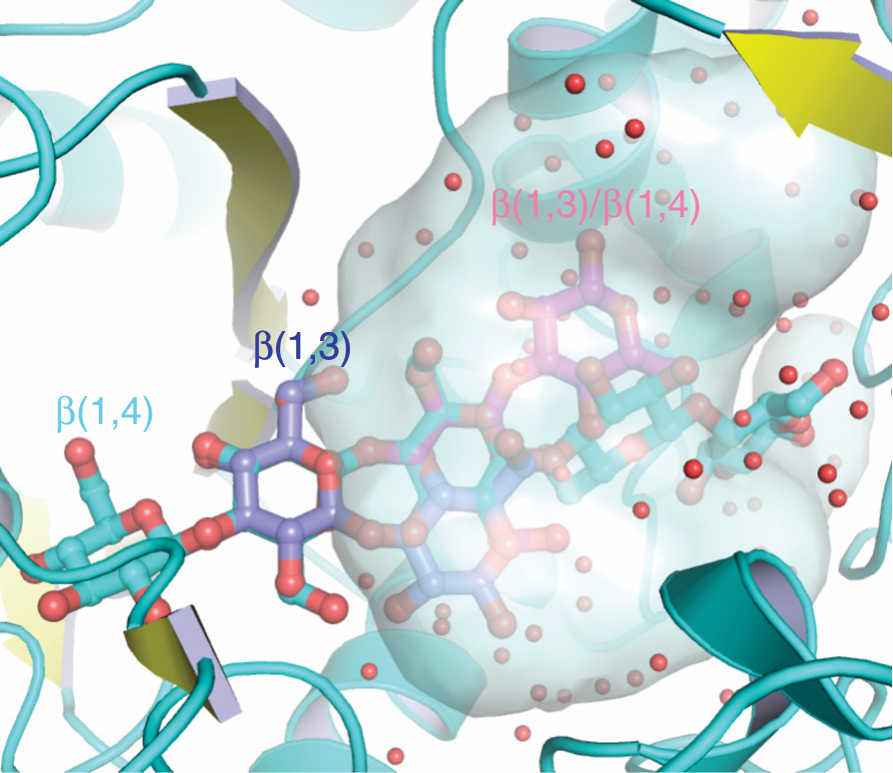
**Carbohydrates accommodated in the tmCBP binding site.** Previous molecular modeling studies of tmCBP identified that in addition to β(1,4) glucosaccharides (cyan), β(1,3) (blue) and mixed β(1,3)/β(1,4) (magenta) could be accommodated in the water-filled (red spheres) non-specific ligand binding subsite. Adapted from [13].

This PBP ligand binding paradigm was recently altered when the crystal structure of the *Thermotoga maritima* cellobiose binding protein (tmCBP) was solved [13]. Unlike other PBPs, the tmCBP binding site is bipartite, being composed of a typical PBP solvent excluded disaccharide binding site (subsite one) that is adjacent to an atypical large solvent filled cavity (subsite two) where three additional saccharide rings could be placed (Figure 1). The structure of tmCBP was solved in the presence of β(1,4) linked sugars, however the size of the tmCBP binding cavity and molecular modeling suggested additional glucan sugar linkages could be accommodated in both the disaccharide binding site and the solvent filled cavity (Figure 1). tmCBP is found in an operon that consists of an ABC transport system and an endoglucanase, the natural substrate of which has been predicted to be the algae-based storage polysaccharide laminarin [32-34]. Using a series of laminarin-based β(1,3) linked carbohydrates we sought to further identify the molecular mechanisms underlying simultaneous encoding of specificity and promiscuity in tmCBP subsites. These studies suggests ways that the bound hydrogen bonding rich water molecules bound in subsite two can potentially be used to adapt and expand ligand binding sites beyond the functionality encoded by the fixed protein scaffold.

## Results and discussion

### Thermal stability and ligand binding specificity of tmCBP

**Figure 2:**
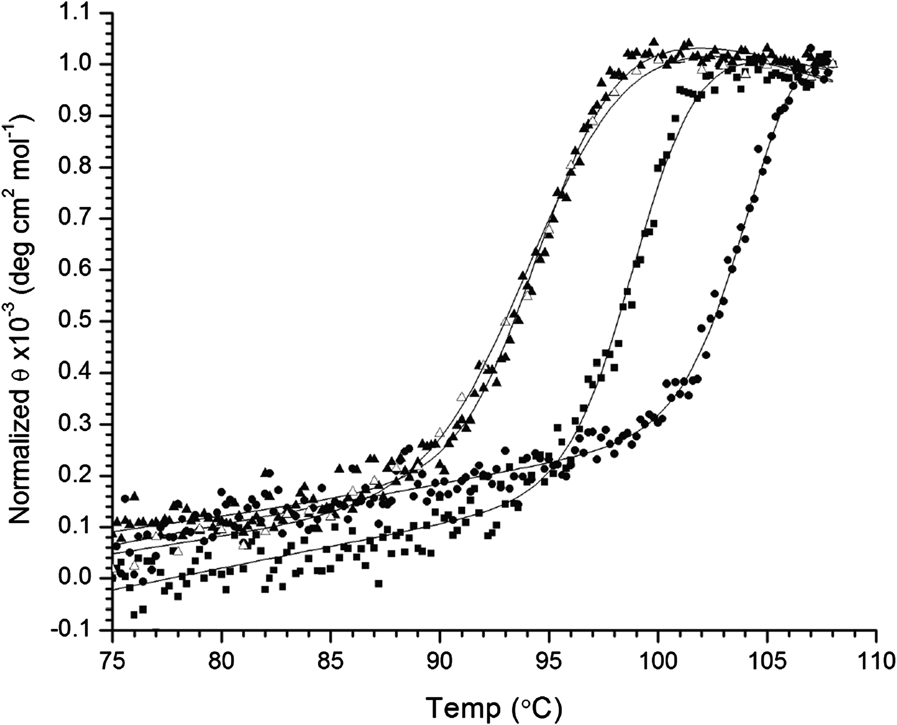
**Thermal denaturation of tmCBP in the presence of laminarin**-**based carbohydrates.** Circular dichroism was used to monitor that thermal denaturation of tmCBP in the absence (solid triangle) and presence of 1 mM laminaribiose (square), laminaripentaose (circle), or xylopentaose (open triangle). Solid lines are a fit to a two-state model for thermal denaturation that takes into account the native and denatured baseline slopes.

Previous molecular modeling of the tmCBP binding [13] site suggested that in addition to β(1,4) oligosaccharides, additional linkages such as β(1,3) carbohydrates or mixed β(1,4)/β(1,3) could be accommodated in the bipartite binding site (Figure 1). Thermal denaturation of tmCBP, monitored by the change in circular dichroism (CD) signal, was used to assess the binding of the xylan-based β(1,4) linked xylose pentasaccharide, xylopentaose, and the laminarin-based β(1,3) linked glucose disaccharide, laminaribiose (LR2), and pentasaccharide, laminaripentaose (LR5). To bring the thermal melting point (T_m_) into a measurable range, experiments were carried out in the presence of the chemical denaturant guanidine hydrochloride at a concentration of 2 M [17,35]. Addition of LR2 and LR5 shifted the T_m_ of the protein from 94.8°C to 99.2°C and 105.2°C respectively (Figure 2). These studies indicate that the tmCBP binding site accommodates β(1,3) glucosaccharides ranging in size from two to five sugar rings, which is consistent with earlier CD binding studies for the β(1,4) glucosaccharides cellobiose and cellopentaose [13]. Unexpectedly, the β(1,4) linked xylose-based sugar, xylopentaose, did not induce a change in the T_m_ indicating a lack of a stabilizing or binding interaction with tmCBP (Figure 2). In the previous molecular modeling of the tmCBP binding site, the first sugar ring of either the β(1,3) or β(1,4) glucosaccharides, and in-turn the C6 hydroxyl, is coincident among the two ligand bound forms (Figure 1). It is likely that the hydrogen bonding interactions of the carbohydrate ring C6 hydroxyl with the protein are important for the discrimination of xylo- and glucosaccharides, as the xylosaccharides lack the C6 carbon and hydroxyl atoms.

### Solution structure of apo and ligand-bound tmCBP

**Figure 3:**
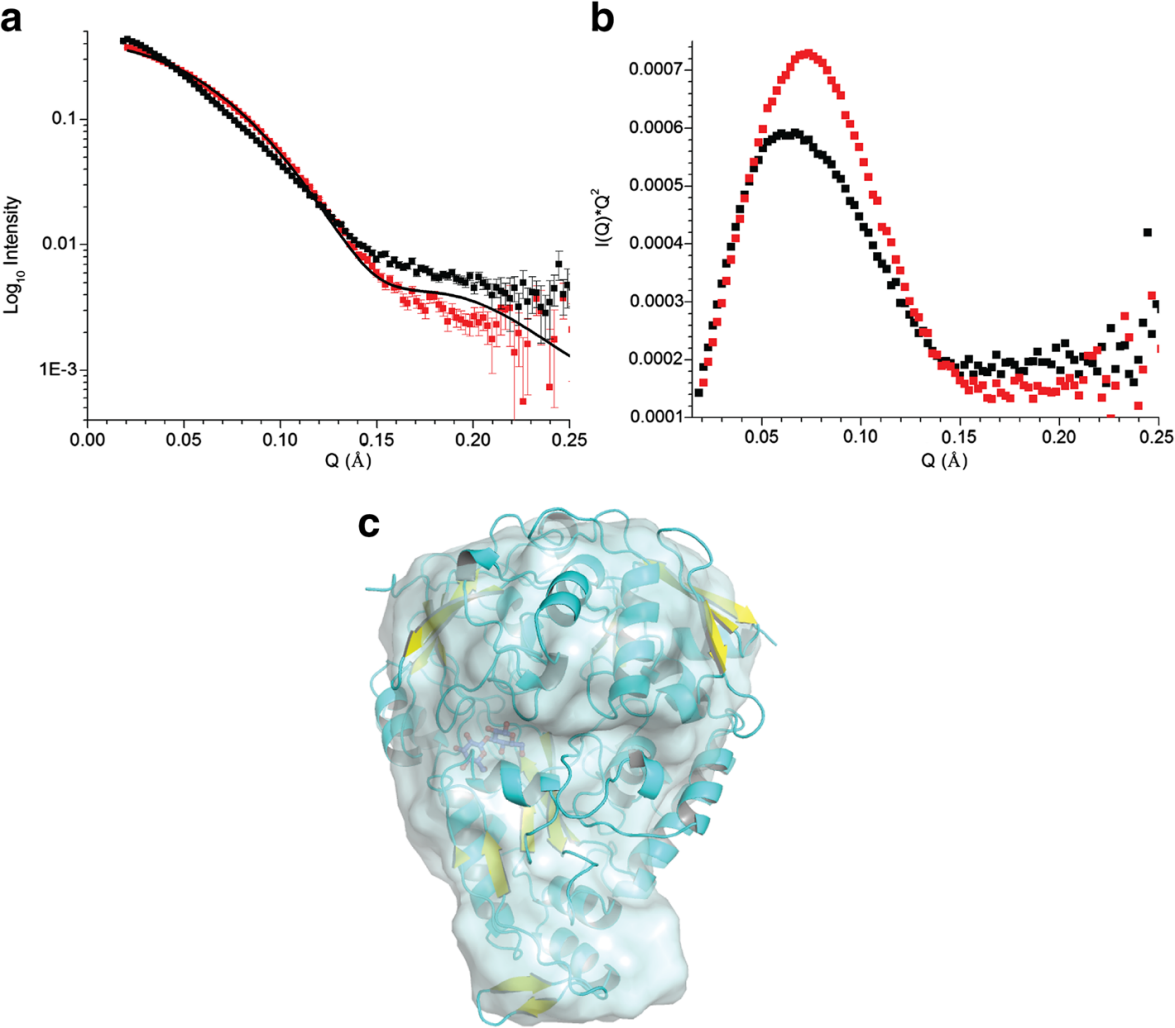
**Small**-**angle neutron scattering of apo and ligand bound tmCBP. (a)** I(q) SANS scattering data of tmCBP in the presence (red squares) and absence (black squares) of 5 mM cellobiose. Solid black line is the CRYSON generated theoretical scattering curve based on the previously determined tmCBP cellobiose complex. **(b)** Krakty plot of apo (black) and cellobiose bound (red) tmCBP. Error bars omitted for clarity. **(c)***Ab*-*initio* model of cellobiose bound tmCBP (surface representation) superimposed with the crystal structure of the cellobiose complex (ribbon representation).

In order to characterize the conformational changes induced upon addition of ligand, small-angle neutron scattering (SANS) data was used to characterize both the apo and ligand bound forms of tmCBP. Cellobiose was used for these studies, which based on the previous crystal structures produces a closed state essentially identical to the laminarin-based carbohydrates. Comparison of the apo and ligand-bound curves show significant differences in both the high q and low q data, indicative of a ligand induced conformational change (Figure 3a). These raw data can be transformed into a Kratky plot where geometrical differences, such as compactness and flexibility, in the scattering particles can be highlighted. In the case of a multi-domain protein connected by flexible linkers, the Kratky plot would show a broad peak at lower q-values, with an upturn at the higher q-values. The Krakty plots for the apo and cellobiose-bound protein are similar in shape and indicative of a globular protein rather than two domains connected by a flexible linker (Figure 3b). This suggests that the tmCBP hinge does not allow for significant conformational flexibility in the absence of ligand which is consistent with the previous small-angle scattering studies of the group II maltose binding protein [36]. It is interesting to speculate as to whether the flexibility of the PBP hinge may be inherent to a particular PBP group as structures of group I PBPs in the absence of ligand have been shown to adopt a series of domain closure angles, perhaps suggesting flexibility in the absence of ligand [29].

Upon addition of ligand the protein undergoes large scale conformational changes as evidenced in the decrease in the radius of gyration and D_max_ (Table 1). The large differences in these biophysical parameters of the scattering particles are of a greater magnitude than one would expect based upon previous small-angle scattering studies of other group II PBPs [36]. Molecular weight determination, based upon the intensity at zero scattering angle suggests that the apo protein forms inter-protein associations that are alleviated upon addition of cellobiose (Table 1). Inter-protein associations have previously been reported for this protein superfamily [37,38]. No molecular modeling of the apo protein was carried out. The SANS data of the cellobiose-bound protein is well accounted for by the previously determined cellobiose-bound tmCBP crystal structure (Figure 3a). This is also observed in comparison of the cellobiose-bound crystal structure and the *ab*-*initio* model generated from the SANS data in the presence of 5 mM cellobiose (Figure 3c).

**Table 1:** SANS data analysis

**Sample**	**Concentration**	**Io**	**Io/C**	**MW (kDa)**		***R***_**g(exp)**_**(Å)**	***R***_**g(calc)**_**(Å)**	***D***_**max**_**(Å)**
	**(mg/mL)**			**Calculated**	**Expected**			
**Apo**	4.2	0.47± 0.001	0.1	73.9	68.7	30.6± 0.04	*	88
	8.4	1.0± 0.001	0.1	73.9	68.7	31.4± 0.03	*	90
	12.5	1.5± 0.002	0.1	73.9	68.7	31.5± 0.03	*	90
	16.7	1.9± 0.002	0.1	73.9	68.7	31.5± 0.015	*	90
**Sat (5 mM Cellobiose)**	4.2	0.39±0.001	0.09	66.5	68.7	24.1± 0.09	23.4	68
	8.4	0.72±0.0007	0.09	66.5	68.7	24.1± 0.02	23.4	67
	12.5	1.05±0.001	0.08	59.1	68.7	24.1± 0.01	23.4	67
	16.7	1.34±0.001	0.08	59.1	68.7	24.0± 0.01	23.4	67

*No model available for apo form.

### Crystal structure of laminaribiose complex

**Figure 4:**
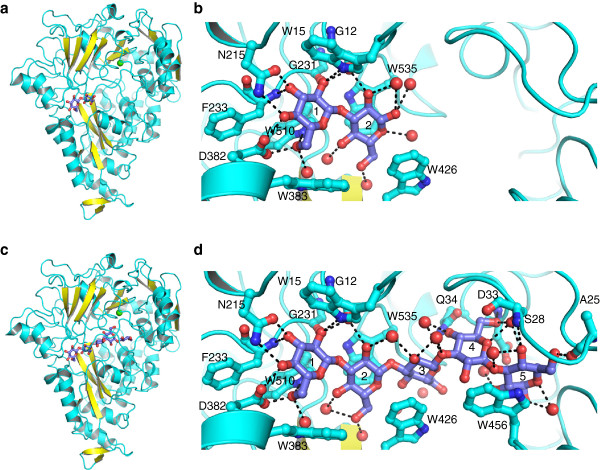
**The X**-**ray crystal structure of ligand bound tmCBP. (a)** Ribbon representation of the overall structure of the laminaribiose bound tmCBP. The laminaribiose ligand is shown in ball and stick representation and the calcium ion is represented as a green sphere. **(b)** Close-up view of the tmCBP amino acids involved in hydrogen bonding (black dashed lines) and van der Waals interaction. The laminaribiose ligand and the amino acids interacting with the ligand are shown in ball and stick representation. **(c)** Ribbon representation of the overall structure of the laminaripentaose bound tmCBP. The laminaripentaose ligand is shown in ball and stick representation and the calcium ion is represented as a green sphere. **(d)** Close-up view of the tmCBP amino acids involved in hydrogen bonding (black dashed lines) and van der Waals interaction. The laminaripentaose ligand and the amino acids interacting with the ligand are shown in ball and stick representation.

The crystal structure of tmCBP complexed with LR2 was solved to a resolution of 2.05 Å by molecular replacement using the previously determined tmCBP structure [13] (Figure 4a). The structure was refined to *R*_work_ and *R*_free_ values of 18.4% and 20.3%, respectively. The final model consists of 582 amino acids, a larminaribiose molecule and 293 water molecules. The overall fold and conformation of the protein is similar to the structure of the cellobiose complexed protein (all atom RMSD= 0.4 Å). Data collection, stereochemistry and refinement statistics are summarized in Table 2.

**Table 2:** Data collection and refinement statistics

	**LR2**	**LR5**
**Data collection**		
Space group	*P*2_1_2_1_2_1_	*P*2_1_2_1_2_1_
Cell parameters (Å)	*a*=56.6 *b*=89.6 *c*=108.2	*a*=56.9 *b*=89.8 *c*=108.3
Resolution range (Å)	50.0-2.05	50.0-2.07
Unique reflections	34613	33022
Redundancy^*a*^	3.4 (3.2)	4.3 (4.2)
Mean *I*/*σ*^*a*^	15.4 (2.5)	16.6 (3.5)
Completeness (%)^*a*^	98.1 (98.3)	95.9 (98.5)
*R*_merge_ (%)^*a*^	6.2 (48.8)	5.2 (49.4)
**Refinement**		
Num. of reflections (working /test set)	32549/2000	30944/1996
*R*_work_/*R*_free_ (%)	18.4/20.3	18.8/22.1
Non-hydrogen atoms in refinement		
Protein	4831	4833
Water	293	237
Metal Ion	1	1
Carbohydrate	23	56
**r.m.s.d.**^***b***^**from ideal**		
Bond lengths (Å)	0.007	0.005
Bond angles (°)	1.7	1.1
**B-factors(Å**^**2**^**)**		
Protein	33.5	38.7
Ligand	26.2	40.9
Metal Ion	28.6	29.0
Solvent	35.8	40.7
**Ramachandran**		
Ramachandran favored (%)	96.0	95.9
Ramachandran allowed (%)	99.5	99.3

^*a*^Number in parentheses represent values in the highest resolution shell.

^*b*^r.m.s.d. indicates root mean square deviation.

An extensive network of polar and non-polar amino acids and water molecules bind the LR2 ligand (Figure 4b). A LigPlot+ representation of the LR2 binding pocket is shown in Additional file 1: Figure S1a [39]. As in other periplasmic carbohydrate binding proteins, a network of aromatic amino acids envelops the ligand between the N- and C-terminal domains. A total of six tryptophan residues (Trp15, Trp380, Trp383, Trp426, Trp510 and Trp535) form van der Waals interactions with the two sugar rings. In total, ten hydrogen bonds can be formed with the first sugar ring. Two hydrogen bonds are formed with the C3 and C4 hydroxyl each, whereas the C2 and C6 hydroxyls form three hydrogen bonds each. All but one hydrogen bond are directly formed with the protein, with the C6 hydroxyl being ligated by a single specifically bound water molecule, W20. For second sugar ring of the laminaribiose, only the C2 hydroxyl forms a direct hydrogen bond with the main chain carbonyl of Gly12, which also forms a hydrogen bond with the C2 hydroxyl of the first ring. The other six hydrogen bonds with the second ring are with water molecules. The C1 hydroxyl of second ring forms hydrogen bonds with specifically bound water molecules, W47 and W150, whereas the C4 and C6 hydroxyls form hydrogen bonds with W39 and W6 water molecules. C2 hydroxyl also forms hydrogen bonds with W47, which forms another hydrogen bond with the main chain carbonyl of Ala13. The O5 hemiacetal oxygen of second ring forms a hydrogen bond with another water molecule, W179 (Figure 4b).

**Figure 5:**
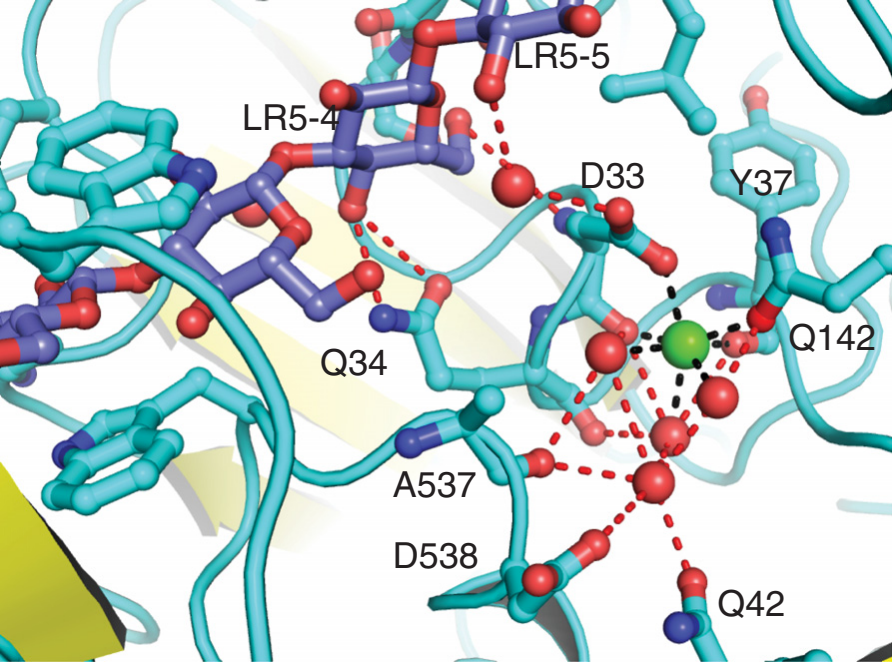
**The tmCBP calcium binding site.** Close-up view of the molecular interactions of tmCBP with an endogenously bound calcium ion (green sphere). Water molecules are shown as red spheres and amino acids involved in hydrogen bonding are represented as ball and stick models. Direct metal hydrogen bonds are represented as black-dashed lines whereas the remainder of the hydrogen bonding network is red. The LR5 crystal structure was used for this analysis.

Unlike the other tmCBP structures that have been solved thus far, both the LR2 complex and the LR5 complex contain a pentagonal bipyrimidally-bound calcium ion (Figures 4a and 5). This calcium ion was bound from the crystallization precipitant solution that contained calcium acetate. The carbonyl of Tyr37, the side-chain Gln142, and the main carbonyl and side chain carboxylate of Asp33 fill four of the seven coordination sites, while the remainders are filled by a network of specifically bound water molecules (Figure 5).

### Crystal structure of laminaripentaose complex

The crystal structure of tmCBP complexed with laminaripentaose (LR5) was solved to 2.07 Å resolution by molecular replacement using the previously determined tmCBP structure [13] (Figure 4c). This structure was refined to *R*_work_ and *R*_free_ values of 18.7% and 22.1%, respectively. The final model consists of 582 amino acids, a laminaripentaose molecule, and 237 water molecules. A calcium ion is also bound in an identical manner as the LR2 complex. The overall fold of the protein is similar to the structure of the cellopentaose complexed protein (all atom RMSD= 0.2 Å). Data collection, stereochemistry and refinement statistics are summarized in Table 2.

Like the LR2 complex, an extensive network of polar and non-polar amino acids and water molecules also bind the LR5 ligand (Figure 4d). A LigPlot+ representation of the LR5 binding pocket is shown in Additional file 1: Figure S1a [39]. The first and second LR5 sugar rings are bound in an identical manner as the LR2 sugar rings. However, The O5 hemiacetal oxygen of the second LR5 sugar ring forms an intra-molecular interaction with the third sugar ring C4 hydroxyl, which replaces the water molecule W179 of LR2. All the hydroxyls of ring 3 of LR5 have only water mediated hydrogen bonds. The C2 hydroxyl and the LR5 ring 3/4 hemiacetal each form two hydrogen bonds with three water molecules, while C4 and C6 hydroxyls form single hydrogen bond with W73 and W15, respectively. Unlike the ring three of LR5, ring four forms direct hydrogen bonds with the protein. The C6 hydroxyl forms two hydrogen bonds, while the C4 hydroxyl and O5 oxygen each form a single hydrogen bond with the protein. An additional water molecule, W129, hydrogen bonds with the C4 hydroxyl of the LR5 ring 4. The C4 hydroxyl of the fifth LR5 ring forms two hydrogen bonds, while the C6 hydroxyl forms a single hydrogen bond with the protein. Three specifically bound water molecules hydrogen bond with the C1, C2 and C4 hydroxyls; two of these waters also separately hydrogen bond with the ring 4/5 O3 hemiacetal oxygen and the O5 oxygen of ring 5. Ring 4 is essentially occupying the positions of W49, W87 and W171 of LR2 complex while ring 5 replaces the W119 water of the LR2 complex.

The calcium ion of the LR5 complex (Figure 4c), with slight variation in coordination distances, has identical coordination geometry as the calcium ion present in the LR2 structure. It is interesting to note that the Asp33, which primarily coordinates the calcium ion also forms hydrogen bonds to C6 hydroxyl of LR5 ring 4 and a water molecule that hydrogen bonds to the C2 hydroxyl of the fifth sugar ring (Figure 5). Moreover, the side chain of Gln34, of which the main chain carbonyl interacts with a water molecule that coordinates with the calcium ion, also forms two hydrogen bonds with the C4 hydroxyl of the LR5 ring four. The localization of this calcium ion suggests it may potentially have a functional role in ligand binding, rather than a structural role as found in other PBPs [22].

### Comparison of laminarin and cellodextrin bound structures

**Figure 6:**
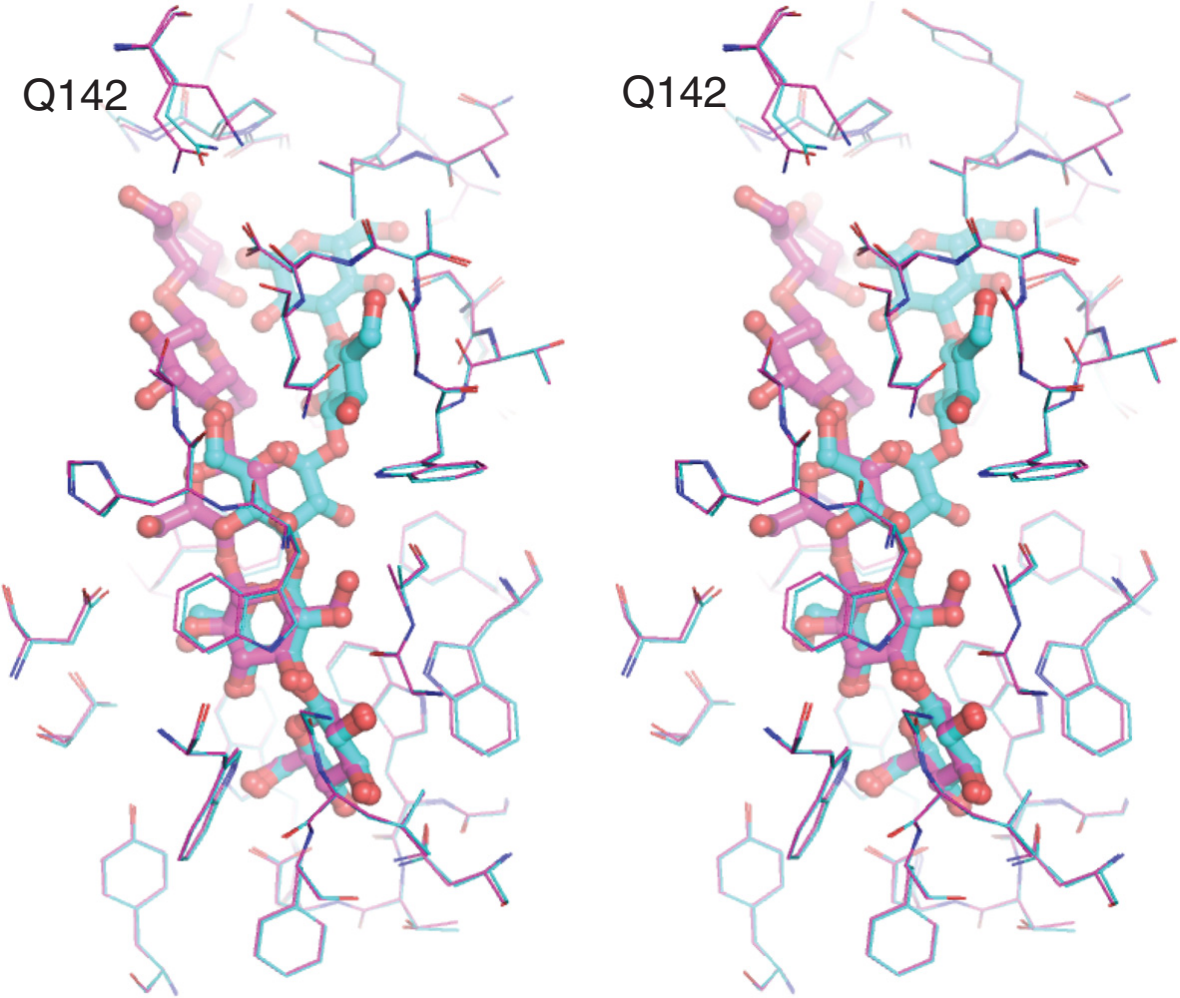
**The tmCBP cellopentaose and laminaripentaose binding site.** Stereo view of the superposition of tmCBP bound with cellopentaose (magenta) and laminaripentaose (cyan). All amino acids that interact with either ligand are shown in line representations.

Comparison of the laminarin and cellodextrin bound tmCBP structures allow for the identification of the molecular details of ligand selectivity in this semi-specific periplasmic binding protein. Superposition of the laminaripentaose (LR5) and cellopentaose (CP5) complexes demonstrates that the conformation of almost every amino acid that forms van der Waals interactions or hydrogen bonds with the LR5 or CP5 in both subsites is in an identical conformation (Figure 6). A single amino acid in subsite two, Gln142, adopts an alternate rotamer between the two structures. As the ligands occupy distinct regions of the binding pocket, which remain fixed in position regardless of which ligand is bound, we postulate that the identical conformation of the hydrogen bonding and van der Waals ligand interaction network suggests the protein binding pocket is pre-ordered for binding of either ligand.

### Role of water molecules in organizing the tmCBP binding site

**Figure 7:**
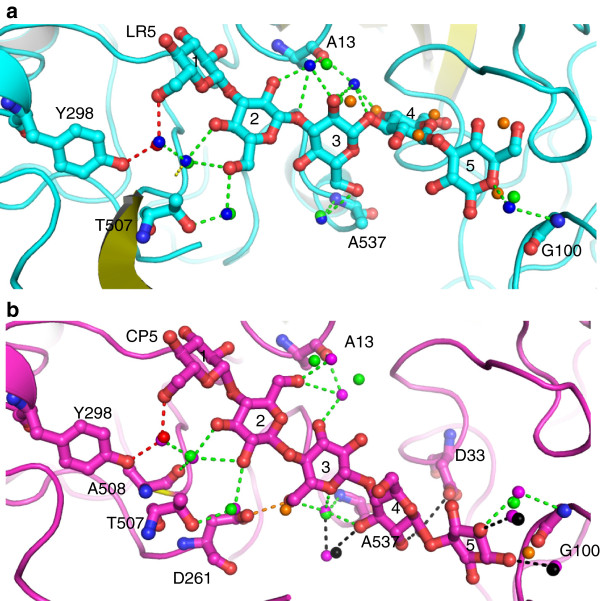
**The network of water molecules modulating the ligand selectivity of tmCBP. (a)** View of the LR5 water network. Water molecules found interacting with the LR5 are shown as blue spheres. The different classes of conserved water molecules also found in the CP5 binding site are colored as follows: red, waters in identical positions coordinating identical atoms; green, waters identical positions coordinating different ligand atoms; black, waters in identical positions forming ligand contacts in one form and not the other; orange, waters mimicking hydroxyl atoms of ligand. **(b)** View of the CP5 water network. Water molecules found interacting with the CP5 are shown as magenta spheres. The different classes of conserved water molecules also found in the LR5 binding site are colored as in **(a)**.

The tmCBP binding site accommodates both β(1,3) and β(1,4) carbohydrates by utilizing the non-specific, subsite two, binding site [13]. Although the ligands occupy different regions of the non-specific subsite, there is significant overlap of the amino acids involved in the recognition of either ligand. We sought to understand how this occurs when the protein amino acid network remains in a fixed conformation. Superposition of the LR5 and CP5 bound structures suggest the bound water network may play an important role in the plasticity of the tmCBP binding site (Figure 7). Five distinct classes of water molecules that are specifically bound to either the N or C-terminal domain are found potentially mediating and modulating ligand selectivity in the tmCBP ligand binding site.

1. *Waters in identical positions coordinating identical atoms on different ligands* (Figure 7, red water molecule): The first LR5 and CP5 sugar rings are in identical positions and the water molecule coordinating the C6 hydroxyl is also found in an identical position. This water simultaneously forms hydrogen bonds with both the N and C-terminal domains of the protein, the C6 hydroxyl of the ligand, and another bound water molecule that interacts with the second sugar ring. As five membered β-xylan carbohydrates do not bind to tmCBP it is possible that the positioning and the bonding network of this water molecule make it important in transducing to the protein that a six membered ring is bound in subsite one. The combination of the lack of this water molecule, and the lacking of the molecular interactions with the C6 carbon and hydroxyl group, potentially impede formation of the closed ligand bound state of the protein in the presence of xylans. These types of bridging, ligand-binding-induced interdomain contacts have been postulated to be important in stimulating the PBP conformational change [27].

2. *Waters in identical positions coordinating different ligand atoms* (Figure 7, green water molecules): Beyond the first sugar ring, the conformation or localization of the sugars in the binding pocket significantly differ. Although coordinating distinct ligand atoms which are located in different regions of subsite two, several water molecules are found conserved in the LR5 and CP5 bound structures. Water molecules that hydrogen bond with ring 2, 3, or 5 of the LR5 ligand are also found in the CP5 bound structure. The conservation of water molecules is also observed in the water molecules interacting with ring 2, 3, or 5 of the CP5 ligand. The rich hydrogen bonding potential of water molecules allows for conservation of water position in both the CP5 and LR5 forms and thereby potentially permits the preordering of the hydrogen bonding potential of subsite one for either type of ligand.

3. *Waters in identical positions forming ligand contacts in one form and not the other* (Figure 7b, black water molecules): Beyond the first two sugar rings found in subsite one, the localization of the LR5 and the CP5 in the tmCBP subsite two differ significantly. However, several water molecules that are involved in forming hydrogen bonds with LR5 are still present when CP5 is bound in subsite two. The same is also true for subsite two waters that hydrogen bond with CP5 and not LR5. This class of water molecule is involved in pre-ordering the rotameric state of subsite two hydrogen bonding residues. Although crystal structure of apo form of tmCBP protein could not be obtained, it is possible the same water-mediated hydrogen bonding network pre-orders subsite 2 for binding of β(1,3) or β(1,4) ligands in the apo state.

4. *Waters that mimic hydroxyls*/*hemiacetals of other ligand* (Figure 7, orange water molecules): Several water molecules are present in either the LR5 or CP5 bound structure that mimic the localization in subsite two of ligand hydroxyl groups of the other ligand. Similar to the class 3 water molecules, this class of water molecules preforms both the water and protein hydrogen bonding interactions for either ligand, the role of which is potentially important for promiscuous ligand recognition of the apo protein.

5. *Secondary shell waters involved in coordinating primary shell waters* (*and*/*or involved coordinating preordering of binding pocket*): Beyond the primary shell water molecules that directly interact with the ligands, a conserved network of at least twelve water molecules is found ordering either the primary shell waters or the amino acids that are involved in interacting with the ligands.

## Conclusions

Depending on biological function, PBPs ligand selectivity is modulated through a combination of binding pocket adaptations that mediate ligand positioning, alter ligand size selection, or alter the free energy of ligand binding in such a manner that excludes incorrect ligands [35,40]. The novel bipartite tmCBP binding site represents an additional, interesting alteration of PBP ligand recognition, exemplifying how both specificity and promiscuity are encoded in a single binding site. Comparison of the tmCBP structures bound to laminarin-based and cellodextrin-based carbohydrates allows for identification of the novel binding selectivity determinants found in this binding site.

The structural changes accompanying ligand binding in PBPs typically involves a re-organization of side-chain rotamers or the protein backbone. Although in some cases one of the two binding half sites in each domain undergoes conformational changes upon ligand binding and this has been suggested as a mechanism of ordering ligand binding [19]. Although lacking the apo crystal structure, analysis of the tmCBP ligand bound state suggests the binding site may be pre-ordered for binding of either laminarins or cellodextrins. Essentially no structural differences are observed among the amino acids in the ligand recognition sphere of either class of ligands. It should be pointed out that no side-chain movement is observed even when the same residue forms direct or indirect interactions with either ligand.

The conservation and positioning of water molecules trapped in the tmCBP binding pocket suggest they potentially play a role in tuning tmCBP ligand selectivity. Several classes of water molecules, playing distinct functional roles, are found in the tmCBP binding site. This network of water molecules preforms the ligand hydrogen bonding network and side chain conformations of ligand interacting amino acids, and thereby reduces the entropic penalty of ligand binding. Additionally, these water molecules are rich in hydrogen bonding potential, allowing for conservation of water placement while facilitating the plasticity of this bipartite binding site.

The mode of ligand binding found in tmCBP represents an interesting adaptation mechanism not previously observed in other PBPs. The downstream carbohydrate transport systems have a narrow, predefined limit to the size and type of carbohydrate that can be processed. The tmCBP binding cavity pre-filters this pool of ligands, potentially optimizing the transport process and in-turn eliminating the energetic penalty of presentation of incorrect carbohydrates to the transport machinery. In *E*. *coli* maltose binding protein ligands that do not fit within the binding site are still bound and presented to the transport machinery [4]. However, with tmCBP a single protein is used to promiscuously select a molecular class of ligands while at the same time sterically restricting the number of rings that can be placed within the constraints of the tmCBP binding cavity. Larger ligands are likely not bound as they would impede the hinge bending motion and in-turn specific ligand recognition.

Seven additional oligosaccharide binding proteins with varying substrate specificities are found in the *T*. *maritima* genome [32]. It remains to be observed whether the mode of ligand recognition in tmCBP is unique or found across this subset of periplasmic carbohydrate binding proteins. The adaptation mechanisms observed in tmCBP allow for expansion of binding site selectivity while maintaining specificity for a molecular class of ligands. These types of adaptation mechanisms could potentially be recapitulated in drug design studies where the rich hydrogen bonding potential of water molecules can be utilized to expand binding sites, or enable multiple drugs to bind to a single target site.

## Methods

### Protein expression and purification

The tmCBP plasmid was transformed into BL21-RIL cells for heterologous expression in either terrific broth media, M9 minimal media or Enfor’s minimal media supplemented with carbennicillin and chloramphenicol. Growth on terrific broth or M9 minimal media produced protein that was bound with a disaccharide ligand as determined by circular dichroism (CD) and X-ray crystallography (data not shown). Ligand-free protein was produced by growth on the glycerol based medium, Enfor’s minimal media. In all cases, tmCBP was purified as previously described [13,32], with slight modifications. Cell pellets were lysed by sonication. The resulting lysate was clarified by centrifugation (34,000 × g) for 20 minutes. Following nickel chelation chromatography purification of the lysate, the protein was loaded on to a Superdex S75 26/60 (Amersham) gel filtration column that was equilibrated with 20 mM Tris, pH 8.0, 150 mM NaCl. This purified material was used for all other experiments.

### Circular dichroism

CD experiments were performed on a Jasco CD spectrophotometer. Thermal denaturations were determined by measuring the CD signal at 225 nm as a function of temperature using 0.5 μM protein in 10mM Tris–HCl pH 8.0 and 40 mM NaCl. In the absence of guanidinium chloride, tmCBP is too stable to exhibit temperature-induced denaturation and all measurements were carried out in the presence of 2 M guanidine hydrochloride and 1.0 mM ligand. CD measurements were fit to a two-state model that takes into account the slope of the native and denatured baselines [41].

### Small angle neutron scattering data collection and analysis

Small-angle neutron scattering (SANS) experiments were performed on the extended Q-range small-angle neutron scattering (EQ-SANS, BL-6) beam line at the Spallation Neutron Source (SNS) located at Oak Ridge National Laboratory (ORNL) [42]. Protein was concentrated to 16.7 mg/mL and dialyzed in to 20 mM Tris pH 8.0, 40 mM NaCl in 100% D_2_O for SANS measurements that were performed at 20°C. Cellobiose was added to the apo protein at a concentration of 5 mM for measurements of the ligand bound form. Data reduction followed standard procedures using MantidPlot (http://www.mantidproject.org/) [43].

Upon verifying a Guinier regime [44] in the SANS profiles, the pair distance distribution function, *P*(*r*), was calculated from the scattering intensity using the indirect Fourier transform method implemented in the GNOM program [45] (Table 1). The real-space radius of gyration, *R*_g_, and scattering intensity at zero angle, *I*(0), were determined from the *P*(*r*) solution to the scattering data. The molecular mass, *M*, was calculated by $I\left(0\right)=M{\left(\Delta \rho \right)}^{2}{\bar{\upsilon }}^{2}/N_{A}$, where Δρ = contrast in scattering length density between protein and D_2_O buffer solution (= ρ_prot_ – ρ_buf_), $\bar{\upsilon }=\text{protein}\;\text{partial}\;\text{specific}\;\text{volume}\;\left(=0.73\;\mathrm{ml}/g\right)$, and *N*_A_ = Avogadro’s number. The GASBOR program [46] was used to generate *ab initio* shape reconstructions.

### Crystallization and X-ray data collection

tmCBP was concentrated to 20 mg∕mL and dialyzed into 10 mM Tris, 40 mM NaCl 0.5 mM TCEP for crystallization. Laminaribiose (LR2) or laminaripentaose (LR5) was added to a final concentration of 1 mM prior to crystallization trials. Crystals were grown by hanging drop vapor diffusion in drops containing 2 μL of the protein solution mixed with 2 μL of 0.2–0.3 M magnesium acetate or calcium acetate, 20–30% (wt/vol) PEG 3350 equilibrated against 900 μL of the same solution. Crystals were transferred to 35% (wt/vol) PEG 3350 for cryoprotection, mounted in a nylon loop, and flash frozen in liquid nitrogen. All X-ray diffraction data were collected at 100 K on a Rigaku 007HFmicromax X-ray generator with a Raxis IV++ detector. The diffraction data were scaled and indexed using HKL3000 [47]. The data collection statistics are listed in Table 2.

### Structure determination, model building and refinement

The LR2 bound and LR5 bound tmCBP structures were solved by molecular replacement using the Phaser program [48]. The crystal structure of the previously determined tmCBP was used as the initial model for fitting the X-ray data ([13], PDB code 3I5O). Manual model building was carried out in COOT [49] and refined using REFMAC5 [48] and PHENIX [50]. The models exhibit good stereochemistry as determined by MolProbity [51]; final refinement statistics are listed in Table 2.

### Accession numbers

Atomic coordinates and structure factors have been deposited in the Protein Data Bank [52] under the accession codes 4JSD and 4JSO for the LR2 complex and LR5 complex, respectively.

## Abbreviations

tmCBP: *Thermotoga maritima* cellobiose binding protein; CD: Circular dichroism; SANS: Small-angle neutron scattering; LR2: Laminaribiose; LR5: Laminaripentaose.

## Competing interests

The authors declare that they have no competing interests.

## Authors’ contributions

MJC, PM and DAM designed the research and drafted the manuscript. MJC and CBC performed small-angle scattering experiments. MJC, PM, SG, and XL performed CD and X-ray crystallography experiments. All authors read and approved the final manuscript.

## Supplementary Material

Additional file 1: Figure S1 Interaction network of tmCBP laminarin ligands. The polar and non-polar contacts of the LR2 (a) and LR5 (b) ligands as generated by LigPlot [39]. The interaction network that is coincident among the LR2 and LR5 structures are highlighted in red, hydrogen bonding interactions are represented as black dashed lines and water molecules as red spheres. Click here for file
