# Supplementary material for: Molecular details of ligand selectivity determinants in a promiscuous β-glucan periplasmic binding protein
Source: BMC Struct Biol. 2013 Oct 4;13:18. doi: 10.1186/1472-6807-13-18 (PMC3850815; doi:10.1186/1472-6807-13-18)
Supplement: Additional file 1: Figure S1 — Interaction network of tmCBP laminarin ligands. The polar and non-polar contacts of the LR2 (a) and LR5 (b) ligands as generated by LigPlot [39]. The interaction network that is coincident among the LR2 and LR5 structures are highlighted in red, hydrogen bonding interactions are represented as black dashed lines and water molecules as red spheres. [file 1472-6807-13-18-S1.pdf]

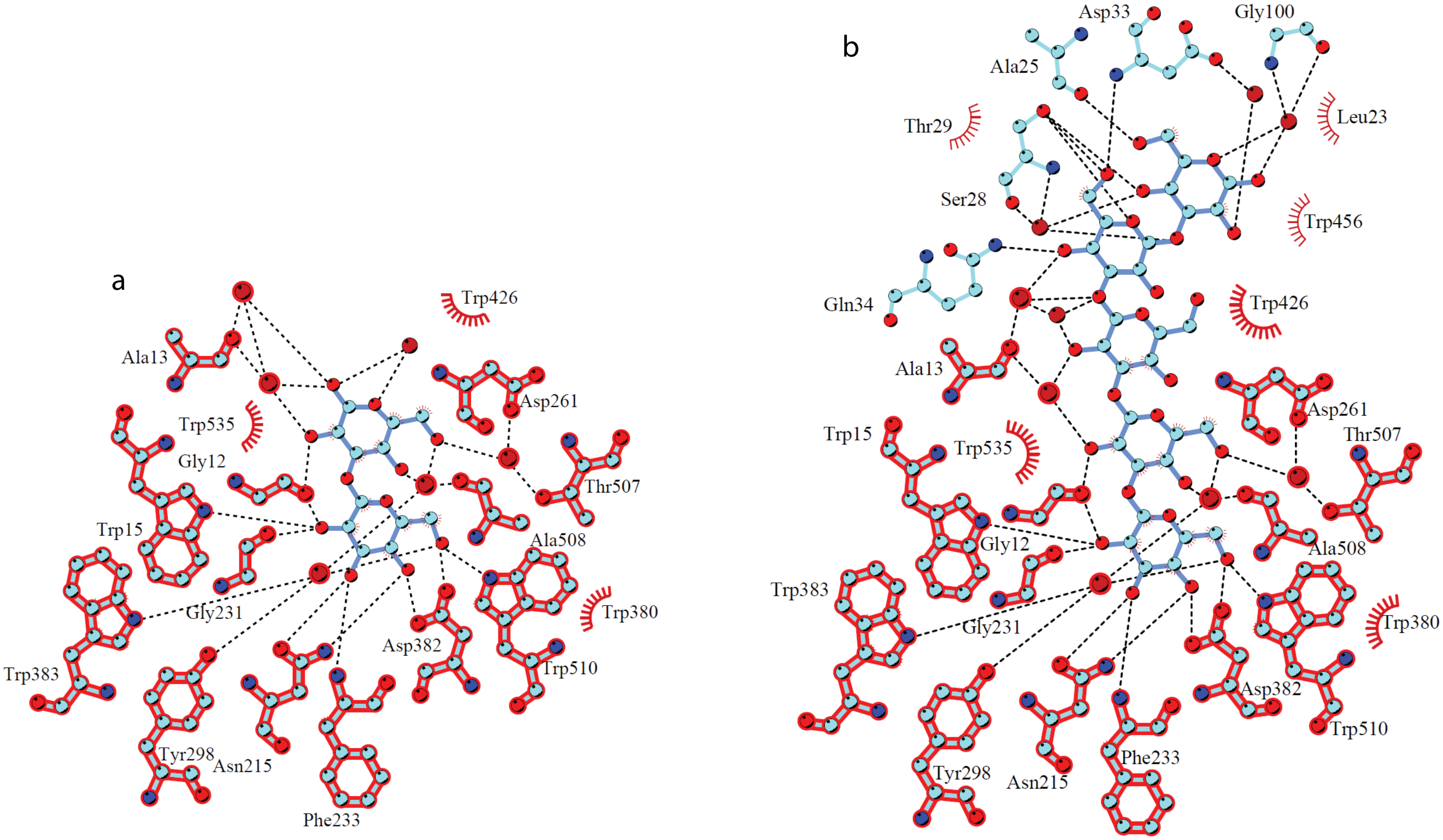

Supplemental Figure 1. Interaction network of tmCBP laminarin ligands. The polar and non-polar contacts of the LR2 (a) and LR5 (b) ligands as generated by LigPlot [39]. The interaction network that is coincident among the LR2 and LR5 structures are highlighted in red, hydrogen bonding interactions are represented as black dashed lines and water molecules as red spheres.
